# Hydration Status and Fluid Replacement Strategies of High-Performance Adolescent Athletes: An Application of Machine Learning to Distinguish Hydration Characteristics

**DOI:** 10.3390/nu13114073

**Published:** 2021-11-15

**Authors:** Haresh T. Suppiah, Ee Ling Ng, Jericho Wee, Bernadette Cherianne Taim, Minh Huynh, Paul B. Gastin, Michael Chia, Chee Yong Low, Jason K. W. Lee

**Affiliations:** 1 Sport and Exercise Science, School of Allied Health, Human Services and Sport, La Trobe University, Bundoora, VIC 3086, Australia; m.huynh@latrobe.edu.au (M.H.); p.gastin@latrobe.edu.au (P.B.G.); 2 National Youth Sports Institute, Singapore 397778, Singapore; eeling.ng@live.com (E.L.N.); jericho_wee@u.nus.edu (J.W.); btaim@research.ait.ie (B.C.T.); low_cheeyong@nysi.org.sg (C.Y.L.); 3 Human Potential Translational Research Programme, Yong Loo Lin School of Medicine, National University of Singapore, Singapore 119228, Singapore; phsjlkw@nus.edu.sg; 4 Department of Sport and Health Sciences, Technological University of the Shannon, Midlands Midwest, Athlone Campus, N37 HD68 Athlone, Ireland; 5 SHE Research Group, Technological University of the Shannon: Midlands Midwest, Athlone Campus, N37 HD68 Athlone, Ireland; 6 Physical Education and Sports Science Group, National Institute of Education, Nanyang Technological University, Singapore 637616, Singapore; michael.chia@nie.edu.sg; 7 Department of Physiology, Yong Loo Lin School of Medicine, National University of Singapore, Singapore 117593, Singapore; 8 Global Asia Institute, National University of Singapore, Singapore 119076, Singapore; 9 N.1 Institute for Health, National University of Singapore, Singapore 117456, Singapore; 10 Institute for Digital Medicine, National University of Singapore, Singapore 117456, Singapore; 11 Singapore Institute for Clinical Sciences, Agency for Science, Technology and Research (A*STAR), Singapore 117609, Singapore

**Keywords:** sport, training intensity, hypohydration, dehydration, young sportsmen/women

## Abstract

There are limited data on the fluid balance characteristics and fluid replenishment behaviors of high-performance adolescent athletes. The heterogeneity of hydration status and practices of adolescent athletes warrant efficient approaches to individualizing hydration strategies. This study aimed to evaluate and characterize the hydration status and fluid balance characteristics of high-performance adolescent athletes and examine the differences in fluid consumption behaviors during training. In total, 105 high-performance adolescent athletes (male: 66, female: 39; age 14.1 ± 1.0 y) across 11 sports had their hydration status assessed on three separate occasions—upon rising and before a low and a high-intensity training session (pre-training). The results showed that 20–44% of athletes were identified as hypohydrated, with 21–44% and 15–34% of athletes commencing low- and high-intensity training in a hypohydrated state, respectively. Linear mixed model (LMM) analyses revealed that athletes who were hypohydrated consumed more fluid (F (1.183.85)) = 5.91, (*p* = 0.016). Additional K-means cluster analyses performed highlighted three clusters: “Heavy sweaters with sufficient compensatory hydration habits,” “Heavy sweaters with insufficient compensatory hydration habits” and “Light sweaters with sufficient compensatory hydration habits”. Our results highlight that high-performance adolescent athletes with ad libitum drinking have compensatory mechanisms to replenish fluids lost from training. The approach to distinguish athletes by hydration characteristics could assist practitioners in prioritizing future hydration intervention protocols.

## 1. Introduction

Maintenance of fluid balance is crucial in optimizing sport performance. Exercise-induced dehydration (i.e., body water deficits exceed 2% of body mass) can hinder endurance performance in warm-hot environments due to increased cardiovascular and thermoregulatory strain [1]. Greater levels of body mass loss (3–4%) have also been recognized to impair cognition, technical skills and physical performance [2]. These fluid deficits are exacerbated when athletes arrive at training in a hypohydrated state [3,4]. Beyond implications on sporting performance, the resultant health outcomes due to dehydration are well documented [5]. These considerations are of importance due to the regularity in which adolescent athletes commence training in a hypohydrated state [6,7]. Evidence also highlights that adolescent athletes often experience voluntary dehydration despite ample fluid availability [6], potentially due to the inability to recognize the signs and symptoms of heat stress [8].

The sweat response of an athlete is influenced by multiple factors, including exercise intensity [9]. Within Australian Rules Football, athletes exhibited greater sweat rates and levels of hypohydration in high-intensity training (1.1–1.3 L/h and 3.4–3.5%) in comparison with low-intensity training (0.8 L/h and 2.1%) [10]. Regardless of the training intensity, fluid balance was not achieved in these athletes. Dehydration, as a result of high-intensity efforts, has been reported to also cause an increase in the subjective ratings of perceived exertion, resulting in potential performance impairments [2].

Research on adults demonstrated that males had greater sweat losses than females, although fluid consumption relative to body mass was similar [11]. However, there is minimal information available on the sex differences of adolescent athletes in sweat loss and fluid replacement. Evidence from collegiate athletes suggest that male athletes consistently displayed higher degrees of dehydration compared to their female counterparts [12]. There is a dearth of studies investigating the effects of sex differences on hydration in the adolescent population. Furthermore, sex differences in adults may not be applicable to adolescents who are still undergoing growth and development [13].

Guidelines have been published to provide practitioners with practical risk stratification and rehydration strategies to individualize fluid requirements based on sport [14]. From an applied sport science support perspective, the differences in athlete hydration status and drinking practices may require more nuanced approaches to characterize athletes into subgroups to individualize rehydration interventions that go beyond the demands of the sport. Novel machine learning techniques for the identification of unique subgroups for targeted healthcare strategies may provide a potential methodology to address this need [15].

Therefore, the general objective of this study was to provide insights on fluid balance and fluid replenishment behaviors during training among heat-acclimatized high-level adolescent athletes training in a warm and humid tropical country. The specific aims of this study were to (i) evaluate and characterize the hydration status and fluid balance characteristics of high-performance adolescent athletes across sports, training intensities and sex; (ii) examine the differences in fluid consumption behaviors during training between euhydrated and hypohydrated athletes; and (iii) cluster the different fluid balance and fluid consumption profiles of high-performance adolescent athletes across multiple sports.

## 2. Materials and Methods

### 2.1. Participants

A total of 199 Singaporean adolescent athletes from 11 sports were recruited via convenience sampling from a high-performance sports academy that identifies and develops talented student-athletes to achieve international sporting success through integrated coaching and sport science services. All participants were highly trained athletes who competed in national and international competitions. Of those initially recruited, a total of 105 participants participated in all the requisite protocols for the entire study and were included in the final analysis. In total, 94 participants were unable to complete all necessary sessions due to academic commitments. Ethical approval for the study was obtained. Demographic data of the 105 participants (male: 66, female: 39) are shown in Table 1.

### 2.2. Design

The design of this field study was descriptive in nature. Hydration status was assessed across three different occasions. First, hydration status was assessed from a first-morning urine sample. The next two occasions were assessed before a ‘low-intensity and ‘high-intensity’ training session to ascertain pre-training hydration status. The research team instructed coaches to differentiate the training sessions based on the expected effort placed on their athletes, with the low-intensity training sessions requiring less relative physical effort than the high-intensity training session and vice versa. To accommodate ecological validity, sessions were planned and executed by the respective coaches for each sport without input from the research team. Participants were blinded to the intended training intensities of each session. The two training sessions were conducted 1 week apart and took place between 1500 and 1800 h.

### 2.3. Procedure

#### 2.3.1. Environmental Conditions

A calibrated handheld environmental meter (Kestrel 5000, Nielsen-Kellerman, Boothwyn, PA, USA) was used to measure the environmental temperature and humidity of each training venue at the start of training. The mean environmental temperature and humidity during the low- and high-intensity sessions were 26.8 ± 3.0 °C, 67 ± 9% and 28.2 ± 4.2 °C, 63 ± 7%, respectively.

#### 2.3.2. Fluid Balance

##### Hydration Status

Hydration status was assessed from the urine samples using urine-specific gravity (USG) and urine color [18]. Hydration status was assessed from first-morning urine samples, and 30 min before the commencement of the low- and high-intensity training sessions. Hydration status was separated into two categories, euhydrated and hypohydrated, based on two urine-based definitions: (i) USG > 1.020 and (ii) USG > 1.025 [17]. The secondary threshold was included for hydration status reporting due to recommendations to raise hypohydration classification thresholds to increase specificity in detecting hypohydration by minimizing false positives [18]. Urine samples were analyzed using a clinical refractometer (model UG-alpha, ATAGO Co., Tokyo, Japan).

Post-training hydration status was determined by changes in body mass, where participants with ≥2.0% body mass change were categorized as dehydrated [19]. Participants were weighed before and after training in minimal attire clad in their training t-shirts and shorts. Swimmers were weighed in their training attire, towel-dried immediately before and after their training. Participants were asked to empty their bladders and defecate if needed before weigh-in. Body mass was measured to the nearest 0.01 kg using a calibrated digital scale (model SECA 874, SECA, Hamburg, Germany).

##### Fluid Consumption

Each participant’s water bottle (empty) was weighed, and refills were done by the investigators for consistency. Refills were made when water bottles were empty. The total volume of fluid in each participant’s water bottle was weighed at the start, and the total number of refills were also recorded. Participants were allowed ad libitum drinking throughout their training. No attempt was made to regulate or influence the hydration behaviors of participants. At the end of their training session, the final mass of each participant’s bottle was measured and recorded. All masses recorded excluded the mass of the bottle and measured to the nearest ±0.01 kg on a digital scale (model ICS241; Mettler Toledo Pte. Ltd., Columbus, OH, USA). The total fluid consumption (per hour) for each participant was calculated based on the following formula:

Fluid consumption (mL) = (Pre-training bottle weight (g) − Post-training bottle weight (g)) + ((Weight of water bottle (filled) − Weight of water bottle (empty)) × Number of refills)
Fluid consumption rate (mL/h) = (Fluid consumption (mL)/Total Training duration (min)) × 60

##### Sweat Rate

Urine output was weighed for sweat rate calculations. Participants were instructed to collect a urine container if they needed to urinate during the training. Bottles were labelled with participant names, and the amount of urine excreted during training was weighed to account for fluid loss by urine excretion. This mass excluded the mass of the container. The sweat rate (per hour) of each participant was calculated by the formula:

Body mass change (g) = Pre-training body mass (g) − Post-training body mass (g)
Sweat loss (mL) = Body mass change (g) + Fluid consumption (mL) − Urine output during training (mL)
Sweat Rate (mL/h) = (Sweat loss (mL)/Total Training duration (min)) × 60

#### 2.3.3. Subjective Measures

##### Training Intensity

Training intensity was determined by obtaining a measure of session Ratings of Perceived Exertion (RPE) using the categorical ratio scale (CR10-scale) [20], recorded at the start and immediately following the end of training.

##### Training Load

Training load was determined by multiplying the end of training session RPE by the duration of the session (in minutes). This product represents, in a single number, the magnitude of internal training load in arbitrary units (AU).

##### Thirst

Thirst sensation was recorded using a 9-point scale, ranging from 1 (not thirsty) to 9 (very thirsty) [21] at the start and immediately following the end of training.

### 2.4. Statistical Analysis

Data analysis was conducted using SPSS Statistics for Windows, version 26 (IBM, Armonk, NY, USA) and R (Version 3.6.1, R Core Development Team), with statistical significance set at *p* < 0.05 with 95% confidence intervals. The results are presented as mean ± SD or frequencies, unless otherwise stated. Linear mixed model (LMM) analyses were used to evaluate differences in fluid consumption (per hour), sweat rate (per hour) and percentage body mass change between training session by sport. The analysis included fixed effects for session x sport. Similarly, LMM analyses were conducted to evaluate the differences in fluid consumption (per hour), sweat rate (per hour) and percentage body mass change between training session by sex, including the fixed effects for session x sex. Sex comparisons were conducted on data from sports with both male and female participants, such that netball and football data were excluded from these analyses. An LMM analysis with fixed effects for pre-training USG hydration classification (based on USG > 1.020 threshold) x session was also employed to examine the differences in fluid consumption behavior between participants with different hydration classifications at the start of training. Participant was included as a random effect for all LMM analysis. The differences in fluid consumption behavior, sweat rate and percentage body mass change by sport and sex were then visualized using a z-score plot. Statistical significance for fixed effects was determined using F tests with the degrees of freedom for F statistics computed using the Satterthwaite approximation method. Bonferroni corrections were made to reduce the likelihood of type-1 error. Effect sizes were calculated using Cohen’s d with the standard errors provided. Effect size thresholds were based on Cohen’s standards and set at 0.2—small, 0.5—medium and 0.8—large [22]. Pearson product-moment correlation coefficients were used to assess relationships between the pre-training USG values and fluid consumption rate, sweat rate and body mass percentage change. A Pearson product-moment correlation coefficient analysis was also conducted to examine relationships between RPE and body mass percentage change. K-means cluster analyses were conducted to identify unique subgroups of drinking characteristics elicited from fluid consumption, sweat rate and percentage body mass change. K-means is an unsupervised machine learning approach to partitioning data that makes no a priori assumptions about the specific variable scores. Analyses for the low- and high-intensity sessions were conducted separately. All variables were transformed into standardized z-scores to standardize the scaling across the variables prior to clustering. The optimal number of cluster solutions (*n* = 3) was chosen based on the elbow method [23]. Cluster assignments were projected onto a 2-dimensional space using Uniform Manifold Approximation and Projection (UMAP).

## 3. Results

### 3.1. Hydration Status and Fluid Balance Characteristics

Hydration status based on first-morning USG analysis classified 20% (using USG > 1.025 classification for hypohydration) and 44% (using USG > 1.020 classification for hypohydration) of the participants as hypohydrated (Table 1).

Pre-training USG analyses showed that 21% (using USG > 1.025 classification for hypohydration) and 44% (using USG > 1.020 classification for hypohydration) of all participants began low-intensity training in a hypohydrated state, while 15% (using USG > 1.025 classification for hypohydration) and 34% (using USG > 1.020 classification for hypohydration) began high-intensity training in a hypohydrated state. In total, 2% of participants in the low-intensity and 0% in the high-intensity session exhibited excessive dehydration, based on ACSM classifications of more than 2% body mass loss [19], following both low- and high-intensity training sessions. The pre- and post-training hydration status are shown in Table 2.

**Figure 1:**
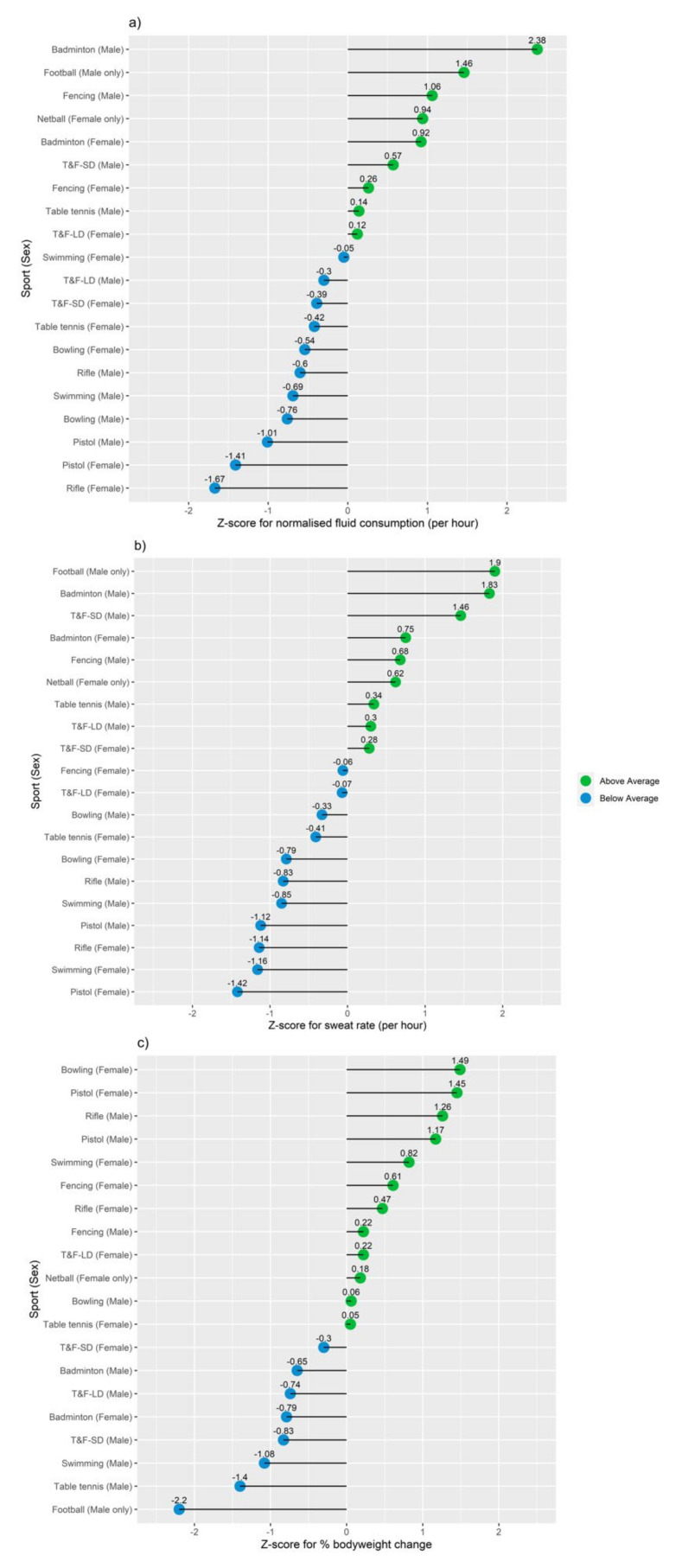
Effects of training on fluid balance and hydration status by sport and sex. (**a**) Z-score plot of fluid consumption (per hour). (**b**) Z-score plot of sweat loss (per hour). (**c**) Z-score plot of percentage body mass change.

Descriptive data on environmental conditions, hydration measures, training intensity and load, thirst, fluid consumption, sweat rate and percentage body mass change pertaining to low- and high-intensity training sessions are provided in Supplementary Materials Table S1. The normalized differences in fluid consumption, sweat rate and body mass percentage change are displayed in Figure 1.

The LMM identified a sport by session interaction for sweat rate (F (10.94) = 2.901, *p* = 0.003) and fluid consumption (per hour) (F (10.94) = 2.297, *p* = 0.018) (Table 3), with post-hoc analyses revealing significant differences between sports on both measures with no differences between sessions (Supplementary Materials Table S2). There was no interaction of sport by session (*p* = 0.173) for percentage body mass change. However, there was a significant main effect of sport (F (10.94) = 3.558, *p* = 0.001) on percentage body mass change, with significant differences between multiple sports (*p* < 0.05; Supplementary Materials Table S3).

There were no sport by gender interactions for sweat rate (*p* = 0.355), fluid consumption (per hour) (*p* = 0.239) or percentage body mass change (*p* = 0.984). Main effect analysis on sex revealed a main effect of sex on sweat rate (F (1.103) = 4.64, *p* = 0.034) and percentage body mass change (F (1.103) = 6.347, *p* = 0.013). Follow-up post-hoc analyses revealed significant differences in the sweat rate (*p* < 0.05) and percentage body mass change (*p* < 0.05) between sexes (Supplementary Materials Table S4).

### 3.2. Fluid Balance Characteristics between Euhydrated and Hypohydrated Athletes

The LMM analysis did not identify an interaction between pre-training USG hydration classification x training session (*p* = 0.881). However, a main effect of pre-training USG hydration classification on fluid consumption (per hour) (F (1.183.85) = 5.91, *p* = 0.016) revealed a significantly higher fluid consumption (per hour) in hypohydrated participants (*p* < 0.05; Supplementary Materials Table S5). The pre-training USG values for all participants revealed a small correlation with fluid consumption (per hour) (R = 0.24; *p* < 0.001) and sweat rate (R = 0.24; *p* < 0.001).

### 3.3. Cluster Analysis-Fluid Balance and Fluid Consumption

**Figure 2:**
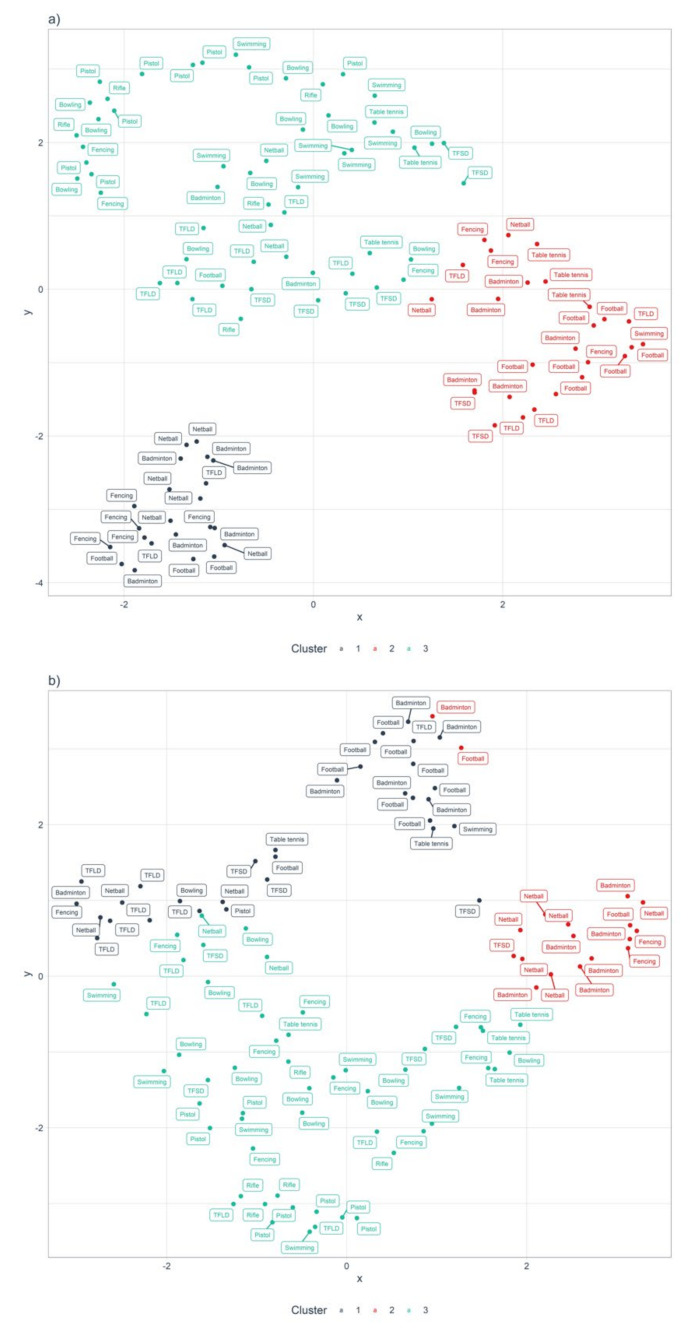
Uniform Manifold Approximation and Projection (UMAP) 2-dimensional projection with k-means cluster assignment. (**a**) Athlete hydration characteristics during low-intensity training. (**b**) Athlete hydration characteristics during high-intensity training. Cluster 1-Heavy sweaters with sufficient compensatory hydration habits (HSSH); Cluster 2-Heavy sweaters with insufficient compensatory hydration habits (HSIH); Cluster 3-Light sweaters with sufficient compensatory hydration habits (LSSH).

The three characteristics (fluid consumption, sweat rate and percentage body mass change) were qualitatively labelled according to the most prominent behaviors in each cluster (Figure 2). Both K-means cluster analyses converged on a three-cluster solution. The clusters were labelled “Heavy sweaters with sufficient compensatory hydration habits” (HSSH), “Heavy sweaters with insufficient compensatory hydration habits” (HSIH) and “Light sweaters with sufficient compensatory hydration habits” (LSSH) (Table 4).

## 4. Discussion

Although several previous studies have examined the hydration status and related variables in adult athletes, there are limited data in high-performance adolescent athletes. To address this gap, the present study aimed to (i) evaluate and characterize the hydration status and fluid balance characteristics of high-performance adolescent athletes across sports, training intensities and sex; (ii) examine the differences in fluid consumption behaviors during training between euhydrated and hypohydrated athletes; and (iii) cluster the different fluid balance and fluid consumption profiles of high-performance adolescent athletes across multiple sports. Overall, our results generally highlight the ability for adolescent athletes in the present study to maintain fluid balance during training, and further, a prevalence of morning and pre-training hypohydration. We also noted differences in the sweat rates and fluid consumption habits between sports, as well as differences in the sweat rates and percentage body mass loss between male and female adolescent athletes.

### 4.1. Hydration Status and Fluid Balance Characteristics

In the present study, the USG definition of >1.020 identified 44% of athletes being hypohydrated in the morning, with 44% and 34%, respectively, commencing low- and high-intensity training in a hypohydrated state. The prevalence of hypohydrated athletes reduced when the USG definition of >1.025 was adopted, with 20% of athletes being hypohydrated in the morning, and 21% and 15%, respectively, commencing low- and high-intensity training in a hypohydrated state. These proportions of morning and pre-training hypohydration are considerably lower than those previously reported in a sample of similarly aged athletes [6,7,24].

Unsurprisingly, we found differences in sweat rates and fluid consumption between sports. Athletes from sports such as pistol, rifle and bowling had significantly lower sweat rates and fluid consumption rates than more physically demanding sports such as badminton and football. The higher sweat rate in badminton compared to several other sports is noteworthy and may be attributed to the high physical demands of the sport [25]. Despite these higher sweat rates, none of the badminton athletes were classified as significantly dehydrated, indicating an adequate fluid intake regime during training. In contrast, our analyses revealed significant reductions in percentage body mass among football (soccer) players in comparison to athletes from several other sports. Whereas the percentage loss in body mass amongst footballers in the current study (~0.7–1.1%) is lower than those reported in previous research in football (>~2%) [2], the percentage of fluid actually replaced during football training in the present study may be indicative of infrequent opportunities rehydrate. Although only a small proportion of the adolescent athletes studied were classified as having experienced dehydration despite the varying levels of sweat loss, this funding should be interpreted with caution as the extent and effects of dehydration may be amplified for athletes that appear for training in a hypohydrated state. Our findings also highlight significantly lower sweat rates and lower percentage body mass loss in females than males. The lower sweat rates in females can be attributed to lower body mass and lower metabolic heat production [26]. There could also be sex differences in sweat gland density [26], sudomotor function [27], hormonal characteristics [28] and thirst perception [29].

### 4.2. Fluid Balance Characteristics between Euhydrated and Hypohydrated Athletes

Another key finding was a habit of higher fluid consumption among athletes that report to training in a hypohydrated state. This might be indicative of thirst-driven drinking behavior that aids in preventing dehydration in adolescent athletes during training. Based on pre-training USG, athletes who arrived at training hypohydrated consumed a significantly higher amount of fluid than those who were euhydrated (mean difference: 82.1 mL/h, 95% CI: 15.5–148.7, *p* < 0.05). It is possible that this self-regulatory drinking behavior minimized the levels of dehydration post-training as body mass losses were within the 2% threshold.

Maresh et al. [30] reported similar results, concluding that extended hypohydration (>3%) preceding low-intensity training stimulates thirst and fluid consumption during exercise in the heat. This increased thirst and associated drinking behavior are likely mediated by hyperosmotic hypovolemia. Whereas the relationships between hypohydration, thirst and drinking appear intuitive, the thirst mechanism is often argued to be an inadequate stimulus to maintain fluid balance. Arnaotis et al. [6] indicated that despite fluid availability, hypohydrated adolescent athletes (pre-training USG > 1.020) were unable to maintain fluid balance by ad libitum drinking. Our results may conflict with those of Arnaotis et al. [6] due to the contextual policy differences in hydration education initiatives present in the current country where the research was conducted [31]. As the training programs in the present study and those within the study by Arnaotis and colleagues [6] were planned and delivered by the respective coaches, differences in breaks or rest periods may have also contributed to the variant fluid balance findings. From a performance perspective, while programmed drinking has been shown to have utility in maintaining fluid balance, it has not conclusively shown to improve performance beyond that of ad libitum drinking in the context of laboratory-based endurance performance [32]. From an applied perspective, rather than adopting a fully ad libitum or programmed drinking schedule to maintain fluid balance, practitioners could employ a decision support strategy, such as one determined by the clustering approach employed in the present study, to determine the optimal support approach for different individuals.

Pre-training hypohydration can increase physiological strain and may exacerbate dehydration [33]. Whereas the underlying mechanisms are unclear, a relationship between hypohydration and increased subjective ratings of perceived exertion have been reported in previous literature on team sport athletes in experimental settings [2]. The present results do not show any associations between the percentage of body mass loss and training intensity. This may be attributed to the field-based observational design of the present study and differences in the modality in which dehydration occurred between sports. The athletes in the present study live in a tropical climate with high temperatures and humidity year-round. Consequently, the importance of drinking water is emphasized through government policy initiatives [31]. Furthermore, fluid availability is further enhanced with the installation of water coolers in all schools [34] and, importantly, the drinkability of municipal tap water [35]. These factors may account for the relatively higher percentage of athletes being euhydrated based on their first-morning and pre-training USG levels in comparison to previous research [6,24].

### 4.3. Cluster Analysis-Fluid Balance and Fluid Consumption

In the present study, a cluster analysis was used to classify athletes based on their underlying hydration characteristics (fluid consumption, sweat rate and percentage body mass change) into three clusters (HSSH, HSIH and LSSH). As evidenced by the number of athletes from different sports within each cluster (Figure 2), such inferences in hydration characteristics may have gone unnoticed without the usage of the k-means unsupervised machine learning technique. Whereas the American College of Sports Medicine [19] recommends the use of an individualized hydration protocol instead of reliance on thirst-driven drinking, the present study highlights the within-sport variability in the sweat responses and drinking behaviors of athletes. This novel approach to hydration monitoring can address the unique behavioral characteristics in hydration practices within each sport and avoid merely providing generic advice or interventional strategies for all athletes within a sport. For example, athletes identified as HSIH may benefit from an individualized drinking strategy to increase fluid intake, such as scheduled drinking breaks and the improvement of hydration accessibility. In contrast, athletes who are classified as LSSH may not require such individualized drinking strategies to increase fluid intake. In such cases, caution should be exercised when encouraging further fluid consumption as it may lead to overhydration and exercise-associated hyponatremia [29]. Instead, practitioners may consider shifting the focus to maintaining thermoregulatory homeostasis to optimize performance while maintaining fluid balance. For instance, athletes who are identified to have adequate self-regulating hydration habits or who are overhydrating may benefit from ice-slurry ingestion as opposed to liquids to reduce the thermal strain from environmental heat and humidity. Indeed, this alternative approach to hydration management can be useful in addressing the widespread belief among adolescent athletes of the need to hydrate more than required due to concerns on the impact on performance [36], especially if there is an incongruence between the athlete’s knowledge and attitudes toward hydration and their actual physiological requirements to maintain fluid balance. Hence, sport practitioners can consider profiling athletes by monitoring their hydration status before and after training, and design interventions accordingly to address the associated health risks and performance effects of exercise-induced hyperthermia due to high ambient temperatures and humidity. Classifying athletes based on hydration status and practice profiles may allow for more efficient and bespoke thermoregulatory support strategies to complement their hydration status and behaviors, especially within large academies or clubs.

Similar machine learning approaches have been used in the clinical setting to identify novel subgroups to individualize treatment regimens in individuals with type 2 diabetes [13]. Future research could examine the impact of tailored treatment strategies, and the inclusion of other considerations (e.g., sodium loss), in improving fluid balance and hydration behavioral outcomes and its impact on sport science support efficiency. With the deluge of data and information currently available within athlete development and sport science [37], the data-informed approach adopted in the present study provides a step toward a more precise, practicality-relevant manner of support stratification and strengthens the utility of decision support systems within a sports science team.

### 4.4. Strengths and Limitations

The main strength of this study was its ability to compare its findings across multiple sports, sex and training intensities in an ecologically valid setting due to the uniform methodology adopted to assess sweat rate, fluid consumption and percentage body mass changes during training in high-performance adolescent athletes. In this regard, the study population was another strength, as the scarcity of research on fluid balance and hydration practices among high-performance adolescent athletes has been acknowledged [8].

The findings of our study should be considered against several limitations. The first was the use of USG instead of plasma osmolality to assess hydration status. The methodological ease and non-invasive nature of this technique make it a viable and widely used protocol in field settings [16]. It is recognized, however, that the use of USG has been documented to result in higher levels of false positives when estimating dehydration [38]. To account for this, we included a secondary threshold of USG > 1.025 when reporting hydration status descriptive data. Finally, due to the applied nature of the study, the classification of low- and high-intensity training sessions was based on the coaches’ interpretation and execution of training plans to elicit the physical efforts necessary for these session types.

## 5. Conclusions

This study provides novel findings on the fluid balance and hydration behaviors of heat-acclimatized high-performance adolescent athletes. In general, adolescent athletes display compensatory strategies to replenish fluid loss during training. However, the prevalence of morning and pre-training hypohydration suggests that efforts need to be placed on non-training centric hydration interventions. Notable differences in hydration characteristics also existed between sport, sex and training intensity. Our results also suggest that high-performance adolescent athletes with ad libitum drinking have compensatory mechanisms to replenish fluids lost from training. Finally, the clustering analysis used in the present study provides an unsupervised machine learning methodology for practitioners to categorize athletes by their inherent sweat rates and hydration practices for a more nuanced management and interventional strategy that goes beyond sport and training intensity.

## Figures and Tables

**Table 1:** Participant demographic and morning hydration values.

		Demographic Data ^1^				USG Values		Hydration Classification ^2^			
	N	Age (Years)	Height (cm)	Mass (kg)	BMI (kg/m^2^)	Morning USG	Morning Urine Color	≤1.020 ^3^	>1.020 ^3^	≤1.025 ^4^	>1.025 ^4^
Badminton	13	14.5 (0.9)	169.2 (10.7)	58.1 (10.7)	20.2 (2.4)	1.019 (0.007)	4.5 (1.9)	8 (61.5)	5 (38.5)	11 (84.6)	2 (15.4)
Bowling	10	14.6 (0.5)	168.9 (8.1)	60.4 (10.0)	21.1 (2.8)	1.020 (0.005)	4.2 (1.9)	6 (60.0)	4 (40.0)	8 (80)	2 (20)
Fencing	11	13.8 (1.2)	168.5 (8.1)	59.5 (13.3)	20.8 (3.8)	1.024 (0.005)	3.7 (1.7)	3 (27.3)	8 (72.7)	7 (63.6)	4 (36.4)
Football	11	13.0 (0.0)	155.1 (5.0)	47.2 (4.6)	19.6 (1.5)	1.021 (0.010)	4.2 (1.9)	4 (36.4)	7 (63.6)	7 (63.6)	4 (36.4)
Netball	11	13.5 (0.5)	165.5 (5.7)	56.1 (10.4)	20.5 (3.5)	1.018 (0.008)	3.5 (2.4)	7 (63.6)	4 (36.4)	9 (81.8)	2 (18.2)
Pistol	9	14.1 (1.4)	163.0 (11.3)	56.0 (11.8)	20.8 (2.2)	1.013 (0.007)	2.8 (2.2)	7 (77.8)	2 (22.2)	8 (88.9)	1 (11.1)
Rifle	5	15.2 (1.1)	165.2 (6.6)	56.9 (9.7)	20.8 (2.9)	1.018 (0.006)	3.6 (1.1)	3 (60.0)	2 (40.0)	5 (100)	0 (0)
Swimming	8	13.5 (0.5)	162.4 (7.9)	52.8 (4.9)	20.1 (1.5)	1.015 (0.006)	3.0 (1.7)	7 (87.5)	1 (12.5)	8 (100)	0 (0)
Table Tennis	6	14.5 (1.4)	167.5 (7.2)	62.2 (3.6)	22.3 (2.3)	1.018 (0.005)	2.5 (0.8)	4 (66.7)	2 (33.3)	6 (100)	0 (0)
Track & Field-LD	13	14.5 (1.1)	166.3 (7.8)	51.5 (8.8)	18.5 (2.4)	1.016 (0.007)	2.5 (1.8)	8 (61.5)	5 (38.5)	11 (84.6)	2 (15.4)
Track & Field-SD	8	14.3 (1.0)	166.5 (7.5)	58.0 (8.6)	20.8 (1.6)	1.024 (0.006)	5.5 (1.8)	2 (25.0)	6 (75.0)	4 (50)	4 (50)
Female	39	14.1 (0.9)	163.6 (7.7)	53.5 (8.3)	20.0 (2.6)	1.018 (0.007)	3.6 (2.0)	23 (59.0)	16 (41.0)	33 (84.6)	6 (15.4)
Male	66	14.1 (1.1)	166.3 (9.2)	57.3 (10.7)	20.6 (2.7)	1.020 (0.007)	3.7 (2.0)	36 (54.5)	30 (45.5)	51 (77.3)	15 (22.7)
Overall	105	14.1 (1.0)	165.3 (8.8)	55.9 (10.0)	20.3 (2.6)	1.019 (0.007)	3.7 (2.0)	59 (56.2)	46 (43.8)	84 (80)	21 (20)

Abbreviations: USG = urine-specific gravity; LD = long distance (>400 m distance events); SD = short distance (≤400 m distance events). Note: ^1^ Data expressed as mean (SD); ^2^ Data presented as *n* (%), where *n* = number of observations across the sport and gender; ^3^ Classifications based on the National Athletic Trainers’ Association position statement [16]; euhydrated ≤ 1.020; hypohydrated > 1.020; ^4^ Classifications based on previous recommendations to raise hypohydration classification thresholds to USG > 1.025 to increase the specificity in detection [17].

**Table 2:** Pre- and post-training hydration status ^1,2^.

		Low Intensity					High Intensity				
		Pre-Training				Post-Training	Pre-Training				Post-Training
	N	≤1.020 ^2^	>1.020 ^2^	≤1.025 ^3^	>1.025 ^3^	Excessive Dehydration ^4^	≤1.020 ^2^	>1.020 ^2^	≤1.025 ^3^	>1.025 ^3^	Excessive Dehydration ^4^
Badminton	13	3 (23.1)	10 (76.9)	8 (61.5)	5 (38.5)	0 (0)	8 (61.5)	5 (38.5)	11 (84.6)	2 (15.4)	0 (0)
Bowling	10	4 (40.0)	6 (60.0)	6 (60)	4 (40)	0 (0)	7 (70.0)	3 (30.0)	8 (80)	2 (20)	0 (0)
Fencing	11	7 (63.6)	4 (36.4)	9 (81.8)	2 (18.2)	0 (0)	3 (27.3)	8 (72.7)	8 (72.7)	3 (27.3)	0 (0)
Football	11	5 (45.5)	6 (54.5)	8 (72.7)	3 (27.3)	1 (9.09)	7 (63.6)	4 (36.4)	9 (81.8)	2 (18.2)	0 (0)
Netball	11	7 (63.6)	4 (36.4)	10 (90.9)	1 (9.1)	0 (0)	8 (72.7)	3 (27.3)	9 (81.8)	2 (18.2)	0 (0)
Pistol	9	9 (100.0)	0 (0)	9 (100)	0 (0)	0 (0)	9 (100.0)	0 (0)	9 (100)	0 (0)	0 (0)
Rifle	5	4 (80.)	1 (20.0)	5 (100)	0 (0)	0 (0)	4 (80.0)	1 (20.0)	5 (100)	0 (0)	0 (0)
Swimming	8	5 (62.5)	3 (37.5)	7 (87.5)	1 (12.5)	1 (12.5)	5 (62.5)	3 (37.5)	7 (87.5)	1 (12.5)	0 (0)
Table Tennis	6	2 (33.3)	4 (66.7)	5 (83.3)	1 (16.7)	0 (0)	4 (66.7)	2 (33.3)	4 (66.7)	2 (33.3)	0 (0)
Track & Field-LD	13	8 (61.5)	5 (38.5)	9 (69.2)	4 (30.8)	0 (0)	10 (76.9)	3 (23.1)	12 (92.3)	1 (7.7)	0 (0)
Track & Field-SD	8	5 (62.5)	3 (37.5)	7 (87.5)	1 (12.5)	0 (0)	4 (50.0)	4 (50.0)	7 (87.5)	1 (12.5)	0 (0)
Female	39	24 (61.5)	15 (38.5)	33 (84.6)	6 (15.4)	0 (0)	30 (76.9)	9 (23.1)	35 (89.7)	4 (10.3)	0 (0)
Male	66	35 (53.0)	31 (47.0)	50 (75.8)	16 (24.2)	2 (3.03)	39 (59.1)	27 (40.9)	54 (81.8)	12 (18.2)	0 (0)
Overall	105	59 (56.2)	46 (43.8)	83 (79.1)	22 (20.9)	2 (1.90)	69 (65.7)	36 (34.3)	89 (84.8)	16 (15.2)	0 (0)

Abbreviations: USG = urine-specific gravity; LD = long distance (>400 m distance events); SD = short distance (≤400 m distance events). Notes: ^1^ Data presented as *n* (%), where *n* = number of observations across the sport and gender by session; ^2^ Classifications based on the National Athletic Trainers’ Association position statement [17]; euhydrated ≤ 1.020; hypohydrated > 1.020; ^3^ Classifications based on previous recommendations to raise hypohydration classification thresholds to USG >1.025 to increase the specificity in detection [18]; ^4^ Classification based on the American College of Sports Medicine position stand guidelines [19]; Excessive dehydration > 2% body mass loss from water deficit.

**Table 3:** Linear mixed model (sport x session intensity) interaction for sweat rate and fluid consumption rate.

	Sweat Rate (mL/h)				Fluid Consumption Rate (mL/h)			
Fixed Effects	Estimates	95% CI ^1^	*p* Value	SMD	Estimates	95% CI ^1^	*p* Value	SMD
Intercept	582	403–761			289	125–454		
Badminton ^a^	151	−75–379	0.19	0.79	288	79–498	<0.05	1.82
Bowling ^a^	−353	−592–−113	<0.01	−1.85	−86	−307–135	0.44	−0.54
Fencing ^a^	109	−126–344	0.36	0.57	234	17–450	0.04	1.47
Football ^a^	284	49–519	<0.05	1.48	273	57–490	<0.05	1.72
Netball ^a^	−51	−286–184	0.67	−0.27	180	−36–397	0.1	1.13
Pistol ^a^	−509	−755–−264	<0.01	−2.67	−182	−408–−44	0.11	−1.15
Rifle ^a^	−348	−636–−60	<0.05	−1.82	−79	−345–186	0.56	−0.50
Swimming ^a^	−394	−647–−141	<0.01	−2.06	−93	−326–140	0.43	−0.58
Table tennis ^a^	−189	−462–84	0.17	−0.99	−63	−315–188	0.62	−0.40
Track & Field-LD ^a^	−17	−244–211	0.89	−0.09	103	−107–312	0.33	0.65
HI ^b^	−35	−224–155	0.72	−0.18	18	−140–176	0.82	0.11
HI*Badminton	156	−85–397	<0.05	0.82	60	−140–260	0.55	0.38
HI*Bowling	192	−62–447	0.14	1.01	7	−204–219	0.95	0.05
HI*Fencing	−263	−512–−13	<0.05	−1.37	−164	−371–43	0.12	−1.03
HI*Football	90	−159–339	0.48	0.47	−53	−260–154	0.61	−0.33
HI*Netball	125	−124–375	0.32	0.66	−26	−233–181	0.81	−0.16
HI*Pistol	119	−142–380	0.37	0.62	48	−169–264	0.66	0.30
HI*Rifle	−27	−333–279	0.86	−0.14	−45	−299–210	0.73	−0.28
HI*Swimming	48	−220–316	0.72	0.25	70	−153–293	0.53	0.44
HI*Table tennis	142	−147–432	0.33	0.75	159	−82–400	0.19	1.00
HI*Track & Field-LD	−145	−386–96	0.24	−0.76	−222	−422–−22	<0.05	−1.4
**Random Effects**								
Between subjects SD	170				174.1			
Within subjects SD	191				158.8			
ICC	0.44				0.55			

Abbreviations: ^1^ 95% CI, 95% confidence interval; SMD, smallest mean difference; LD, long distance (>400 m distance events). Notes: ^a^ reference group = Track & Field-SD (≤400 m distance events), ^b^ reference condition = low-intensity session.

**Table 4:** Final cluster centres (standardized Z values) and mean values (in brackets) of fluid balance and fluid consumption variables.

	Low-Intensity			High-Intensity		
	(Cluster 1)	(Cluster 2)	(Cluster 3)	(Cluster 1)	(Cluster 2)	(Cluster 3)
Variable	Heavy Sweaters with Sufficient Compensatory Hydration Habits	Heavy Sweaters with Insufficient Compensatory Hydration Habits	Light Sweaters with Sufficient Compensatory Hydration Habits	Heavy Sweaters with Sufficient Compensatory Hydration Habits	Heavy Sweaters with Insufficient Compensatory Hydration Habits	Light Sweaters with Sufficient Compensatory Hydration Habits
Fluid consumption rate	1.49 (811.87 mL/h)	−0.20 (311.66 mL/h)	−0.49 (227.22 mL/h)	1.60 (769.85 mL/h)	−0.07 (343.68 mL/h)	−0.5 (233.22 mL/h)
Sweat rate	1.05 (886 mL/h)	0.48 (678 mL/h)	−0.65 (261 mL/h)	0.83 (770 mL/h)	0.68 (720 mL/h)	−0.72 (267 mL/h)
Percentage body mass loss	0.65 (0.03%)	−1.24 (−1.05%)	0.34 (−0.15%)	0.70 (0.02%)	−1.07 (−1.12%)	0.45 (−0.15%)

## Data Availability

The data presented in this study are available on request from the corresponding author. The data are not publicly available due to privacy and ethical issues.

## Supplementary Materials

The following are available online at https://www.mdpi.com/article/10.3390/nu13114073/s1, Table S1.1: Low-intensity training measures, Table S1.2: High-intensity training measures, Table S2.1: Pairwise comparison between sports for sweat rate, Table S2.2: Pairwise comparison between sessions for sweat rate, Table S2.3: Pairwise comparison between sports for fluid consumption rate, Table S2.4: Pairwise comparison between sessions for fluid consumption rate, Table S3.1: Main effect of sport on percentage body mass change, Table S3.2: Pairwise comparison between sports for percentage body mass change, Table S4.1: Main effects of gender on sweat rate and percentage body mass change, Table S4.2: Pairwise comparison between genders for sweat rate, Table S4.3: Pairwise comparison between genders for percentage body mass change, Table S5.1: Main effect of pre-training USG hydration classification on fluid consumption rate, Table S5.2: Pairwise comparison between pre-training USG hydration classification for fluid consumption rate.
