# Supplementary material for: Hydration Status and Fluid Replacement Strategies of High-Performance Adolescent Athletes: An Application of Machine Learning to Distinguish Hydration Characteristics
Source: Nutrients. 2021 Nov 15;13(11):4073. doi: 10.3390/nu13114073 (PMC8625246; doi:10.3390/nu13114073)
Supplement: Supplementary file 1 [file nutrients-13-04073-s001.zip › Supplementary material 1.pdf]

Supplementary material 1

Table S1.1. Low-intensity training measures.

	Environment		Pre-training hydration		Training									
	**Temperature (°c)**	**Humidity (%)**	**USG**	**Urine Colour**	**Pre-training RPE**	**Post-training RPE**	**Duration (mins)**	**Training Load**	**Pre-training Thirst Level**	**Post-training Thirst Level**	**Fluid consumption rate (ml/h)**	**Percentage of fluid replaced (%)**	**Sweat rate (ml /h)**	**Percentage body mass change (%)**
Badminton	26.1 (0.0)	69 (0)	1.024 (0.005)	5.4 (2.0)	3.2 (1.6)	5.4 (2.3)	104.2 (20.8)	577 (283)	3.2 (1.6)	5.2 (0.9)	578 (400)	81 (22)	733 (400)	-0.4 (0.4)
Bowling	23.3 (0.0)	67 (0)	1.020 (0.009)	2.9 (1.5)	2.5 (1.2)	4.5 (1.6)	105.6 (1.9)	476 (177)	3.8 (1.7)	3.7 (1.9)	203 (118)	114 (86)	229 (118)	0.0 (0.5)
Fencing	23.0 (0.0)	51 (0)	1.017 (0.009)	3.3 (2.0)	1.5 (1.3)	4.8 (1.8)	66.0 (40.8)	348 (276)	2.5 (1.3)	4.1 (2.2)	523 (307)	9 (223)	691 (307)	-0.3 (0.5)
Football	32.9 (0.0)	62 (0)	1.020 (0.007)	5.1 (2.2)	0.4 (0.8)	4.6 (1.5)	90.0 (0.0)	414 (136)	2.7 (1.3)	4.6 (1.7)	563 (406)	64 (32)	866 (406)	-0.7 (1.1)
Netball	29.5 (0.0)	70 (0)	1.013 (0.009)	1.8 (1.5)	3.2 (1.7)	4.7 (1.3)	87.5 (9.7)	407 (103)	3.2 (1.4)	4.9 (1.6)	469 (229)	86 (30)	531 (229)	-0.2 (0.4)
Pistol	25.4 (0.0)	63 (0)	1.007 (0.005)	1.4 (1.3)	3.1 (1.3)	3.2 (1.1)	65.0 (0.0)	209 (71)	4.7 (1.9)	5.7 (1.8)	107 (169)	197 (264)	73 (170)	0.0 (0.3)
Rifle	25.4 (0.0)	63 (0)	1.015 (0.007)	2.4 (1.9)	3.6 (2.3)	4.6 (1.7)	68.0 (7.6)	313 (123)	5.6 (1.7)	6.0 (1.2)	210 (133)	159 (153)	234 (133)	0.0 (0.3)
Swimming	29.7 (0.0)	63 (0)	1.017 (0.008)	3.4 (1.6)	3.5 (1.2)	4.4 (2.0)	105.0 (0.0)	465 (209)	3.4 (2.4)	3.6 (2.3)	197 (145)	187 (238)	188 (145)	-0.7 (0.8)
Table Tennis	26.1 (0.0)	69 (0)	1.020 (0.007)	2.0 (1.5)	2.5 (1.0)	6.4 (1.5)	180.0 (0.0)	1152 (273)	3.3 (1.8)	4.0 (1.9)	226 (74)	59 (163)	393 (74)	-0.8 (0.4)
Track & Field-LD	25.2 (0.0)	81 (0)	1.019 (0.008)	4.6 (2.3)	3.2 (2.0)	4.7 (1.6)	58.8 (5.5)	273 (86)	3.8 (1.7)	4.5 (1.7)	392 (200)	77 (35)	565 (200)	-0.3 (0.4)
Track & Field-SD	28.1 (0.0)	80 (0)	1.018 (0.007)	3.5 (2.3)	4.8 (2.1)	5.5 (1.3)	60.0 (0.0)	338 (71)	5.3 (1.6)	6.5 (0.9)	289 (161)	48 (23)	582 (161)	-0.5 (0.2)
Female	26.9 (2.3)	69 (8)	1.015 (0.009)	2.6 (2.1)	3.2 (1.7)	4.8 (1.5)	87.9 (30.8)	427 (227)	3.8 (1.8)	4.9 (1.8)	321 (229)	103 (112)	398 (245)	-0.2 (0.4)
Male	26.7 (3.3)	66 (9)	1.019 (0.008)	4.0 (2.2)	2.6 (1.9)	4.8 (1.8)	87.1 (34.3)	425 (283)	3.5 (1.8)	4.7 (1.8)	401 (326)	87 (151)	560 (415)	-0.4 (0.6)
Overall	26.8 (3.0)	67 (9)	1.018 (0.008)	3.5 (2.2)	2.8 (1.8)	4.8 (1.7)	87.4 (32.9)	426 (263)	3.6 (1.8)	4.8 (1.8)	371 (295)	93 (138)	500 (369)	-0.3 (0.6)

Abbreviations: USG = Urine specific gravity; LD = Long distance (> 400 m distance events), SD = Short distance (≤ 400 m distance events)

Note: Data expressed as mean (SD).

Table S1.2. High-intensity training measures.

	Environment		Pre-training hydration		Training									
	Temperature (∘c)	Humidity (%)	USG	Urine Colour	Pre-training RPE	Post-training RPE	Duration (mins)	Training Load	Pre-training Thirst Level	Post-training Thirst Level	Fluid consumption rate (ml/h)	Percentage of fluid replaced (%)	Sweat rate (ml/h)	Percentage body mass change (%)
Badminton	30.1 (0.0)	66 (0)	1.018 (0.009)	3.4 (2.4)	3.1 (1.3)	8.5 (1.6)	126.9 (13.2)	1093 (279)	3.5 (1.6)	6 (1.5)	656 (290)	79 (29)	8557 (326)	-0.6 (0.9)
Bowling	23.7 (0.0)	66 (0)	1.016 (0.009)	2.8 (1.6)	3.3 (1.7)	4.9 (1.4)	77.4 (2.7)	380 (117)	4.6 (1.9)	5 (2.2)	228 (96)	62 (27)	387 (101)	-0.3 (0.3)
Fencing	23 (0.0)	51 (0)	1.020 (0.009)	4.4 (2.5)	0.8 (0.8)	4.9 (1.4)	109.1 (13.6)	545 (192)	3.7 (1.2)	4.1 (1.8)	377 (258)	98 (61)	393 (135)	-0.2 (0.6)
Football	34.7 (0.0)	52 (0)	1.015 (0.009)	3.6 (2.4)	0.6 (1.1)	5.1 (1.6)	82.0 (0.0)	418 (135)	2.7 (1.1)	5.8 (1.7)	527 (211)	58 (23)	921 (205)	-1.1 (0.6)
Netball	29.3 (0.0)	70 (0)	1.013 (0.01)	2.6 (2.4)	3.3 (1.9)	6.2 (1.4)	63.3 (5.6)	386 (72)	3.5 (1.7)	5.9 (1.5)	462 (321)	68 (36)	622 (135)	-0.4 (0.4)
Pistol	26.3 (0.0)	68 (0)	1.009 (0.007)	1.7 (1.7)	3.7 (1.6)	3.8 (2.5)	96.7 (10.9)	360 (219)	4.0 (1.7)	3.7 (1.6)	173 (102)	225 (221)	157 (164)	0.1 (0.5)
Rifle	23.6 (0.0)	59 (0)	1.014 (0.006)	3.0 (0.7)	3.2 (1.9)	5.6 (2.1)	90.0 (0.0)	504 (187)	5.0 (1.9)	6.6 (0.5)	184 (101)	114 (48)	173 (111)	0.0 (0.2)
Swimming	31.0 (0.0)	61 (0)	1.018 (0.006)	3.0 (1.8)	4.1 (2)	6.4 (2.3)	120.0 (0.0)	765 (272)	3.0 (1.6)	4.4 (2.6)	285 (210)	66 (74)	201 (248)	-0.3 (0.5)
Table Tennis	19.9 (0.0)	64 (0)	1.020 (0.008)	4.0 (2.3)	3.0 (1.1)	6.0 (1.8)	135.0 (0.0)	810 (242)	4.0 (1.1)	4.3 (1.9)	403 (99)	82 (20)	501 (121)	-0.4 (0.4)
Track & Field-LD	30.5 (0.0)	70 (0)	1.012 (0.009)	2.5 (1.7)	3.1 (2.5)	4.3 (2.5)	102.7 (16.9)	454 (266)	3.4 (1.6)	4.5 (1.9)	188 (165)	70 (106)	386 (338)	-0.6 (0.8)
Track & Field-SD	31.7 (0.0)	61 (0)	1.017 (0.008)	2.6 (1.4)	5.0 (2.5)	4.9 (1.4)	60.0 (0.0)	293 (81)	4.8 (1.8)	5.8 (2.0)	307 (129)	56 (20)	547 (151)	-0.4 (0.2)
Female	28.1 (3.4)	65 (5)	1.013 (0.009)	2.5 (20.)	3.6 (1.9)	5.5 (2.2)	88.8 (29.2)	503 (327)	4.1 (1.7)	5.1 (2.1)	346 (235)	94 (103)	433 (244)	-0.3 (0.5)
Male	28.2 (4.6)	61 (7)	1.017 (0.009)	3.3 (2.0.)	2.5 (2.1)	5.5 (2.2)	101.0 (21.2)	575 (299)	3.5 (1.6)	5.1 (1.9)	370 (269)	82 (84)	540 (360)	-0.5 (0.7)
Overall	28.2 (4.2)	63 (7)	1.015 (0.009)	3.0 (2.0)	2.9 (2.1)	5.5 (2.2)	96.4 (25)	547 (310)	3.7 (1.6)	5.1 (1.9)	361 (256)	87 (91)	501 (324)	-0.4 (0.6)

Abbreviations: USG = Urine specific gravity; LD = Long distance, SD = Short distance

Note: Data expressed as mean (SD).
