# Supplementary material for: Hydration Status and Fluid Replacement Strategies of High-Performance Adolescent Athletes: An Application of Machine Learning to Distinguish Hydration Characteristics
Source: Nutrients. 2021 Nov 15;13(11):4073. doi: 10.3390/nu13114073 (PMC8625246; doi:10.3390/nu13114073)
Supplement: Supplementary file 1 [file nutrients-13-04073-s001.zip › Supplementary material 2.pdf]

Supplementary material 2

Table S2.1. Pairwise comparison between sports for sweat rate.

(I) Sport	(J) Sport	Mean difference (I-J) (ml/h)	SE	*P* value[a]	95% CI for difference (ml/h)
Badminton	Bowling	485.9**	91.4	< 0.01	172.6–799.2
	Fencing	252.0	89.1	0.31	-53.1–557.1
	Football	-99.4	89.1	1.00	-404.5–205.8
	Netball	217.8	89.1	0.90	-87.3–522.9
	Pistol	679.1**	94.3	< 0.01	356.2–1002.1
	Rifle	590.4**	114.4	< 0.01	198.5–982.4
	Swimming	599.3**	97.7	< 0.01	264.6–934
	Table tennis	347.3	107.3	0.09	-20.3–714.9
	Track & Field-LD	318.3*	85.3	< 0.05	26.1–610.4
	Track & Field-SD	229.4	97.7	1.00	-105.3–564.1
Bowling	Fencing	-233.9	95.0	0.86	-559.3–91.5
	Football	-585.2**	95.0	< 0.01	-910.7– -259.8
	Netball	-268.1	95.0	0.32	-593.5–57.3
	Pistol	193.2	99.9	1.00	-149–535.5
	Rifle	104.5	119.1	1.00	-303.4–512.5
	Swimming	113.4	103.1	1.00	-239.9–466.7
	Table tennis	-138.6	112.3	1.00	-523.2–246.1
	Track & Field-LD	-167.6	91.4	1.00	-480.9–145.7
	Track & Field-SD	-256.5	103.1	0.80	-609.8–96.8
Fencing	Football	-351.4*	92.7	< 0.05	-668.9– -33.8
	Netball	-34.2	92.7	1.00	-351.8–283.4
	Pistol	427.1**	97.7	< 0.01	92.4–761.9
	Rifle	338.4	117.2	0.27	-63.3–740.1
	Swimming	347.3	101.0	0.05	1.2–693.4
	Table tennis	95.3	110.3	1.00	-282.7–473.3
	Track & Field-LD	66.3	89.1	1.00	-238.9–371.4
	Track & Field-SD	-22.6	101.0	1.00	-368.7–323.5
Football	Netball	317.1	92.7	0.05	-0.5–634.7
	Pistol	778.5**	97.7	< 0.01	443.7–1113.2
	Rifle	689.8**	117.2	< 0.01	288.1–1091.5
	Swimming	698.7**	101.0	< 0.01	352.6–1044.7
	Table tennis	446.7*	110.3	< 0.05	68.7–824.7
	Track & Field-LD	417.6**	89.1	< 0.01	112.5–722.7
	Track & Field-SD	328.7	101.0	0.09	-17.3–674.8
Netball	Pistol	461.3**	97.7	< 0.01	126.6–796.1
	Rifle	372.6	117.2	0.11	-29.1–774.4
	Swimming	381.5*	101.0	< 0.05	35.5–727.6
	Table tennis	129.5	110.3	1.00	-248.5–507.5
	Track & Field-LD	100.5	89.1	1.00	-204.6–405.6
	Track & Field-SD	11.6	101.0	1.00	-334.5–357.7
Pistol	Rifle	-88.7	121.3	1.00	-504.1–326.7
	Swimming	-79.8	105.6	1.00	-441.7–282.1
	Table tennis	-331.8	114.6	0.26	-724.3–60.7
	Track & Field-LD	-360.9*	94.3	< 0.05	-683.8–-37.9
	Track & Field-SD	-449.7**	105.6	< 0.01	-811.6–-87.8
Rifle	Swimming	8.9	123.9	1.00	-415.7–433.5
	Table tennis	-243.1	131.6	1.00	-694.1– 207.9

	Track & Field-LD	-272.2	114.4	1.00	-664.1– 119.8
	Track & Field-SD	-361.0	123.9	0.25	-785.6–63.6
Swimming	Table tennis	-252.0	117.4	1.00	-654.2–150.3
	Track & Field-LD	-281.1	97.7	0.27	-615.7–53.6
	Track & Field-SD	-369.9	108.7	0.05	-742.3–2.5
Table tennis	Track & Field-LD	-29.1	107.3	1.00	-396.7–338.5
	Track & Field-SD	-117.9	117.4	1.00	-520.2–284.3
Track & Field-LD	Track & Field-SD	-88.9	97.7	1.00	-423.5–245.8

Abbreviations: SE, standard error; 95% CI, 95% confidence interval; LD = Long distance (> 400 m distance events), SD = Short distance (≤ 400 m distance events)

Notes: Based on estimated marginal means; *The mean difference is significant at the .05 level; **The mean difference is significant at the .01 level; [a]Adjustment for multiple comparisons: Bonferroni.

Table S2.2. Pairwise comparison between sessions for sweat rate.

(I) Session	(J) Session	Mean Difference (I-J) (ml/h)	SE	*P* value[a]	95% CI for difference (ml/h)
High intensity	Low intensity	5.4	27.51	0.845	-49.2–60.0

Abbreviations: SE, standard error; 95% CI, 95% confidence interval

Notes: Based on estimated marginal means; [a]Adjustment for multiple comparisons: Bonferroni.

Table S2.3. Pairwise comparison between sports for fluid consumption rate.

(I) Group	(J) Group	Mean Difference (I-J) (ml/h)	SE	*P* value[a]	95% CI for difference (ml/h)
Badminton	Bowling	401.0**	87.2	< 0.01	102.4–699.7
	Fencing	166.8	84.9	1.00	-124–457.7
	Football	71.7	84.9	1.00	-219.2–362.6
	Netball	151.1	84.9	1.00	-139.7–442
	Pistol	476.4**	89.9	< 0.01	168.5–784.3
	Rifle	419.8*	109.0	< 0.05	46.2–793.4
	Swimming	375.9*	93.1	< 0.05	56.8–694.9
	Table tennis	302.2	102.3	0.22	-48.2–652.6
	Track & Field-LD	326.4*	81.3	< 0.05	47.9–604.9
	Track & Field-SD	318.3	93.1	0.05	-0.7–637.3
Bowling	Fencing	-234.2	90.5	0.62	-544.4–76
	Football	-329.3*	90.5	< 0.05	-639.5–-19.1
	Netball	-249.9	90.5	0.38	-560.1–60.3
	Pistol	75.4	95.2	1.00	-250.8–401.6
	Rifle	18.8	113.5	1.00	-370.1–407.6
	Swimming	-25.1	98.3	1.00	-361.9–311.6
	Table tennis	-98.8	107.0	1.00	-465.5–267.8
	Track & Field-LD	-74.6	87.2	1.00	-373.2–224
	Track & Field-SD	-82.7	98.3	1.00	-419.5–254
Fencing	Football	-95.1	88.4	1.00	-397.9–207.6
	Netball	-15.7	88.4	1.00	-318.5–287
	Pistol	309.6	93.1	0.07	-9.5–628.7
	Rifle	253.0	111.8	1.00	-130–635.9
	Swimming	209.1	96.3	1.00	-120.8–539
	Table tennis	135.4	105.2	1.00	-225–495.7
	Track & Field-LD	159.6	84.9	1.00	-131.3–450.5
	Track & Field-SD	151.5	96.3	1.00	-178.4–481.4
Football	Netball	79.4	88.4	1.00	-223.3–382.1
	Pistol	404.7**	93.1	< 0.01	85.6–723.8
	Rifle	348.1	111.8	0.13	-34.9–731
	Swimming	304.2	96.3	0.12	-25.7–634.1
	Table tennis	230.5	105.2	1.00	-129.9–590.8
	Track & Field-LD	254.7	84.9	0.19	-36.2–545.6
	Track & Field-SD	246.6	96.3	0.66	-83.3–576.5
Netball	Pistol	325.3*	93.1	< 0.05	6.2–644.4
	Rifle	268.7	111.8	1.00	-114.3–651.6
	Swimming	224.8	96.3	1.00	-105.1–554.7
	Table tennis	151.1	105.2	1.00	-209.3–511.4
	Track & Field-LD	175.3	84.9	1.00	-115.6–466.2
	Track & Field-SD	167.2	96.3	1.00	-162.7–497.1
Pistol	Rifle	-56.6	115.6	1.00	-452.6–339.4
	Swimming	-100.5	100.7	1.00	-445.5–244.5
	Table tennis	-174.2	109.2	1.00	-548.4–200
	Track & Field-LD	-150.0	89.9	1.00	-457.8–157.9
	Track & Field-SD	-158.1	100.7	1.00	-503.1–186.9
Rifle	Swimming	-43.9	118.1	1.00	-448.7–360.9
	Table tennis	-117.6	125.5	1.00	-547.5–312.3
	Track & Field-LD	-93.4	109.0	1.00	-467–280.3
	Track & Field-SD	-101.5	118.1	1.00	-506.3–303.3
Swimming	Table tennis	-73.7	111.9	1.00	-457.1–309.7

	Track & Field-LD	-49.5	93.1	1.00	-368.5–269.6
	Track & Field-SD	-57.6	103.6	1.00	-412.6–297.4
Table tennis	Track & Field-LD	24.2	102.3	1.00	-326.2–374.6
	Track & Field-SD	16.1	111.9	1.00	-367.3–399.5
Track & Field-LD	Track & Field-SD	-8.1	93.1	1.00	-327.2–310.9

Abbreviations: SE, standard error; 95% CI, 95% confidence interval; LD = Long distance (> 400 m distance events), SD = Short distance (≤ 400 m distance events)

Notes: Based on estimated marginal means; *The mean difference is significant at the .05 level; **The mean difference is significant at the .01 level; [a]Adjustment for multiple comparisons: Bonferroni.

Table S2.4. Pairwise comparison between sessions for fluid consumption rate.

(I) Session	(J) Session	Mean Difference (I-J) (ml/h)	SE	*P* value[a]	95% CI for difference (ml/h)
High intensity	Low intensity	2.9	22.9	0.90	-42.5–48.4

Abbreviations: SE, standard error; 95% CI, 95% confidence interval

Notes: Based on estimated marginal means; [a]Adjustment for multiple comparisons: Bonferroni.
