# Supplementary material for: Hydration Status and Fluid Replacement Strategies of High-Performance Adolescent Athletes: An Application of Machine Learning to Distinguish Hydration Characteristics
Source: Nutrients. 2021 Nov 15;13(11):4073. doi: 10.3390/nu13114073 (PMC8625246; doi:10.3390/nu13114073)
Supplement: Supplementary file 1 [file nutrients-13-04073-s001.zip › Supplementary material 3.pdf]

Supplementary material 3

Table S3.1. Main effect of sport on percentage body mass change.

Fixed effects	Estimates	95% CI	*P* value	SMD
Intercept	-0.44	-0.76– -0.12		
Badminton [a]	-0.07	-0.48–0.33	0.72	-0.15
Bowling [a]	0.26	-0.16– 0.69	0.23	0.54
Fencing [a]	0.22	-0.2–0.63	0.31	0.44
Football [a]	-0.50	-0.92– -0.08	< 0.05	-1.03
Netball [a]	0.17	-0.24–0.59	0.41	0.36
Pistol [a]	0.49	0.05–0.92	0.03	1.01
Rifle [a]	0.43	-0.08–0.94	0.10	0.89
Swimming [a]	-0.05	-0.5–0.4	0.83	-0.1
Table tennis [a]	-0.14	-0.62–0.35	0.58	-0.28
Track & Field-LD [a]	-0.02	-0.43–0.38	0.90	-0.05
**Random effects**				
Between subjects SD	0.29			
Within subjects SD	0.48			
ICC	0.27			

Abbreviations: 95% CI, 95% confidence interval; SMD, smallest mean difference; LD, Long Distance (> 400 m distance events), SD = Short distance (≤ 400 m distance events)

Note: [a] reference group = Track & Field-SD (≤ 400 m distance events).

Table S3.2. Pairwise comparison between sports for percentage body mass change.

(I) Sport	(J) Sport	Mean difference (I-J)	SE	*P* value[a]	95% CI for difference (%)
Badminton	Bowling	-0.34	0.19	1.00	-0.99–0.32
	Fencing	-0.29	0.19	1.00	-0.92–0.34
	Football	0.42	0.19	1.00	-0.21–1.06
	Netball	-0.25	0.19	1.00	-0.88–0.39
	Pistol	-0.56	0.20	0.28	-1.23–0.11
	Rifle	-0.51	0.24	1.00	-1.32–0.31
	Swimming	-0.03	0.20	1.00	-0.72–0.67
	Table tennis	0.06	0.22	1.00	-0.7–0.83
	Track & Field-LD	-0.05	0.18	1.00	-0.66–0.56
	Track & Field-SD	-0.07	0.20	1.00	-0.77–0.62
Bowling	Fencing	0.05	0.20	1.00	-0.63–0.72
	Football	0.76*	0.20	< 0.05	0.08–1.44
	Netball	0.09	0.20	1.00	-0.59–0.76
	Pistol	-0.23	0.21	1.00	-0.94–0.48
	Rifle	-0.17	0.25	1.00	-1.02–0.68
	Swimming	0.31	0.21	1.00	-0.43–1.04
	Table tennis	0.40	0.23	1.00	-0.4–1.2
	Track & Field-LD	0.29	0.19	1.00	-0.37–0.94
	Track & Field-SD	0.26	0.21	1.00	-0.47–1
Fencing	Football	0.71*	0.19	< 0.05	0.05–1.37
	Netball	0.04	0.19	1.00	-0.62–0.70
	Pistol	-0.27	0.20	1.00	-0.97–0.42
	Rifle	-0.22	0.24	1.00	-1.05–0.62
	Swimming	0.26	0.21	1.00	-0.46–0.98
	Table tennis	0.35	0.23	1.00	-0.43–1.14
	Track & Field-LD	0.24	0.19	1.00	-0.39–0.87
	Track & Field-SD	0.22	0.21	1.00	-0.5–0.93
Football	Netball	-0.67*	0.19	< 0.05	-1.33– -0.01
	Pistol	-0.99**	0.20	< 0.01	-1.68– -0.29
	Rifle	-0.93*	0.24	< 0.05	-1.77– -0.1
	Swimming	-0.45	0.21	1.00	-1.17–0.27
	Table tennis	-0.36	0.23	1.00	-1.15–0.42
	Track & Field-LD	-0.47	0.19	0.66	-1.11–0.16
	Track & Field-SD	-0.50	0.21	1.00	-1.22–0.22

Netball	Pistol	-0.32	0.20	1.00	-1.01–0.38
	Rifle	-0.26	0.24	1.00	-1.1–0.57
	Swimming	0.22	0.21	1.00	-0.5–0.94
	Table tennis	0.31	0.23	1.00	-0.48–1.09
	Track & Field-LD	0.20	0.19	1.00	-0.44–0.83
	Track & Field-SD	0.17	0.21	1.00	-0.55–0.89
Pistol	Rifle	0.06	0.25	1.00	-0.81–0.92
	Swimming	0.54	0.22	0.90	-0.22–1.29
	Table tennis	0.62	0.24	0.56	-0.19–1.44
	Track & Field-LD	0.51	0.20	0.57	-0.16–1.18
	Track & Field-SD	0.49	0.22	1.00	-0.26–1.24
Rifle	Swimming	0.48	0.26	1.00	-0.4–1.36
	Table tennis	0.57	0.27	1.00	-0.37–1.51
	Track & Field-LD	0.46	0.24	1.00	-0.36–1.27
	Track & Field-SD	0.43	0.26	1.00	-0.45–1.32
Swimming	Table tennis	0.09	0.24	1.00	-0.75–0.92
	Track & Field-LD	-0.02	0.20	1.00	-0.72–0.67
	Track & Field-SD	-0.05	0.23	1.00	-0.82–0.73
Table tennis	Track & Field-LD	-0.11	0.22	1.00	-0.88–0.65
	Track & Field-SD	-0.14	0.24	1.00	-0.97–0.7
Track & Field-LD	Track & Field-SD	-0.03	0.20	1.00	-0.72–0.67

Abbreviations: SE, standard error; 95% CI, 95% confidence interval; LD = Long distance (> 400 m distance events), SD = Short distance (≤ 400 m distance events)

Notes: Based on estimated marginal means; *The mean difference is significant at the .05 level; **The mean difference is significant at the .01 level; [a]Adjustment for multiple comparisons: Bonferroni.
