# Supplementary material for: Hydration Status and Fluid Replacement Strategies of High-Performance Adolescent Athletes: An Application of Machine Learning to Distinguish Hydration Characteristics
Source: Nutrients. 2021 Nov 15;13(11):4073. doi: 10.3390/nu13114073 (PMC8625246; doi:10.3390/nu13114073)
Supplement: Supplementary file 1 [file nutrients-13-04073-s001.zip › Supplementary material 4.pdf]

Supplementary material 4

Table S4.1. Main effects of gender on sweat rate and percentage body mass change.

	Sweat rate (per hour)				Percentage body mass change (%)			
**Fixed effects**	**Estimates**	**95% CI**	***P* value**	**SMD**	**Estimates**	**95% CI**	***P* value**	**SMD**
Intercept	415.6	317.4 – 513.8			-0.23	-0.39 – -0.07		
Male [a]	134.6	10.7 – 258.5	< 0.05	0.65	-0.25	-0.45 – -0.05	< 0.05	-0.52
**Random effects**								
Between subjects SD	272.5				0.35			
Within subjects SD	206.8				0.48			
ICC	0.63				0.35			

Abbreviations: 95% CI, 95% confidence interval; SMD, smallest mean difference

Notes: [a] reference group = Female.

Table S4.2. Pairwise comparison between genders for sweat rate.

(I) Gender	(J) Gender	Mean Difference (I-J) (ml/h)	SE	*P* value[a]	95% CI for difference (ml/h)
Male	Female	134.6*	62.5	< 0.05	10.7 – 258.5

Abbreviations: SE, standard error; 95% CI, 95% confidence interval

Notes: Based on estimated marginal means; *The mean difference is significant at the .05 level; [a]Adjustment for multiple comparisons: Bonferroni.

Table S4.3. Pairwise comparison between genders for percentage body mass change.

(I) Gender	(J) Gender	Mean Difference (I-J) (%)	SE	*P* value[a]	95% CI for difference (%)
Male	Female	-0.25*	0.10	< 0.05	-0.45 – -0.05

Abbreviations: SE, standard error; 95% CI, 95% confidence interval

Notes: Based on estimated marginal means; *The mean difference is significant at the .05 level; [a]Adjustment for multiple comparisons: Bonferroni.
