# Supplementary material for: Hydration Status and Fluid Replacement Strategies of High-Performance Adolescent Athletes: An Application of Machine Learning to Distinguish Hydration Characteristics
Source: Nutrients. 2021 Nov 15;13(11):4073. doi: 10.3390/nu13114073 (PMC8625246; doi:10.3390/nu13114073)
Supplement: Supplementary file 1 [file nutrients-13-04073-s001.zip › Supplementary material 5.pdf]

Supplementary material 5

Table S5.1. Main effect of pre-training USG hydration classification on fluid consumption rate.

Fixed effects	Estimates (ml/h)	95% CI (ml/h)	*P* value	SMD
Intercept	334.2	280.6 – 387.7		
Hypohydrated[a]	82.1	15.5 – 148.7	< 0.05	0.49
**Random effects**				
Between subjects SD	210.9			
Within subjects SD	168.8			
ICC	0.61			

Abbreviations: 95% CI, 95% confidence interval; SMD, smallest mean difference
Notes: [a] reference group = Euhydrated.

Table S5.2. Pairwise comparison between pre-training USG hydration classification for fluid consumption rate.

(I) USG status	(J) USG status	Mean Difference (I-J) (ml/h)	SE	*P* value[a]	95% CI for difference (ml/h)
Hypohydrated	Euhydrated	82.1*	33.76	< 0.05	15.5 – 148.7

Abbreviations: SE, standard error; 95% CI, 95% confidence interval

Notes: Based on estimated marginal means; *The mean difference is significant at the .05 level; [a]Adjustment for multiple comparisons: Bonferroni.
